# Substance-induced manic psychosis in which delusions were corroborated by a chatbot - case report

**DOI:** 10.1186/s12888-026-08137-3

**Published:** 2026-06-04

**Authors:** Sachin Shah, Hamilton Morrin

**Affiliations:** 1 St George’s Hospital Psychiatry Liaison Service, South West London and St George’s Mental Health NHS Trust, London, UK; 2 Department of Psychological Medicine, Institute of Psychiatry, Psychology & Neuroscience, King’s College London, London, UK; 3 Neuropsychiatry Service, South London and Maudsley NHS Foundation Trust, London, UK

**Keywords:** Substance-induced psychosis, Mania, Artificial intelligence, Large language models, Chatbots, Delusions, Digital mental health, Risk management, Case report

## Abstract

**Background:**

This case describes a substance-induced manic episode with psychotic features in which interaction with an AI (artificial intelligence) chatbot appeared to corroborate and reinforce the patient’s delusional thought content and to contradict medical advice. Excerpts from the patient’s interactions with the AI chatbot provide novel clinical insight into this phenomenon, which to date has primarily been reported in news media.

**Case presentation:**

A man in his 30s presented to the emergency department with a one-week history of escalating behavioural disturbance, severe insomnia, pressured and overinclusive speech, and grandiose beliefs. Symptom onset followed heavy polysubstance use at a recreational event, including psilocybin (dried mushrooms and liquid preparation), ketamine, cocaine, and alcohol. During this period, the patient reported extensive interaction with an AI chatbot (ChatGPT). The AI chatbot reportedly affirmed his perceived “spiritual awakening,” minimised the possibility that his presentation represented a manic episode, and provided medical advice, including discouragement of prescribed antipsychotic medication, though it cannot be determined to what extent, if any, these statements contributed to his existing presentation. Mental state examination was consistent with a manic episode with psychotic features, without evidence of perceptual disturbance. He was detained under mental health legislation for further assessment and commenced on olanzapine, with adjunctive sleep restoration and psychological interventions. Behavioural management included implementation of a care plan restricting AI chatbot use, as a form of environmental containment. Over several weeks, psychotic symptoms and behavioural disinhibition diminished, with subsequent improvement in insight.

**Conclusions:**

Concerns regarding potentially harmful interactions between AI chatbots and individuals with mental illness have largely been raised in news media. This case demonstrates that, in patients with psychotic symptoms, AI chatbots may reinforce delusional beliefs and impair the development of insight, and may also interfere with engagement with treatment by providing advice that conflicts with clinical recommendations. These observations raise clinical, ethical, and risk-management considerations regarding AI chatbot use during acute psychiatric illness. As AI chatbot use becomes increasingly widespread, clinicians should consider assessing their use and impact within clinical assessments and, where clinically indicated, implementing interventions to mitigate associated risks, ranging from psychoeducation to use-restriction strategies. Future population-level studies are required to establish the epidemiology of AI-associated mental health harms, and AI companies must bolster efforts to implement harm minimisation strategies and safeguards.

## Background

Digital tools are increasingly used by patients to appraise symptoms and seek health-related guidance [1–3]. An October 2025 survey of UK adults found over a third had used an AI chatbot for support with their mental health [4], and figures from OpenAI suggest that 0.07% of weekly users (or 560,000 individuals) show signs of psychosis or mania each week [5], though in both cases results have not been peer reviewed, and self-report [4] and automated analyses [5] may impact the validity of findings. AI chatbots powered by large language models (LLMs) are designed to generate fluent and personalised responses [6, 7] and can be as persuasive as humans, with persuasive performance likely being shaped by contextual factors such as LLM model, conversation design and domain [8]. AI chatbots can be sycophantic (i.e. exhibit excessive flattery or agreement with users), and this can increase attitude extremity and overconfidence [9], and decrease prosocial behaviour and encourage dependence on said chatbots [10].

Delusions are understood as being shaped by an interplay of cognitive and affective processes [11] and have been proposed to evolve over a temporally extended, embodied and cognitive-linguistic trajectory characterised by an emotional transformation of the self and world [12]. In psychiatry, there is the historical and rare phenomenon of shared psychotic disorder (or *folie à deux*) where one individual transmits their delusional beliefs to a second party, often with lower epistemic authority [13, 14]. With vulnerable users, sycophantic AI chatbots may inadvertently validate delusional ideation or offer advice that conflicts with clinical care plans [5, 15–18], and this process has been likened to a technological *folie à deux* [16], or “emotional contagion” in cases of mania [19]. Reports describing AI-mediated amplification of psychopathology remain limited; however, early case narratives suggest that sycophantic AI chatbots exhibiting excessive flattery or agreement with users [9] may mirror, validate or amplify delusional beliefs, including grandiosity, messianic ideation and transformational narratives [15, 16, 19–25].

One previously published clinical case report describes psychosis following bromide ingestion that was reportedly suggested by an AI chatbot [26], whilst another describes a medical professional with depression, anxiety, and ADHD who developed delusional beliefs that she was interacting with her deceased relative through ChatGPT [25].

This case contributes to existing literature by describing the temporal sequence and content of a patient’s protracted AI chatbot interaction during a substance-induced manic episode with psychotic features, and by outlining practical management strategies (for example, a device care plan restricting AI chatbot access) that were associated with clinical improvement.

## Case presentation

A white male in his 30s with no prior psychiatric history voluntarily attended the emergency department, accompanied by his partner and his friend, who were concerned after he had displayed approximately seven days of abnormal behaviour following a recreational event at which he had ingested psilocybin (approximately 10 g of dried mushrooms and 5 ml of liquid preparation), ketamine (approximately 1 g), cocaine (two lines), and alcohol (four beers). Following the event, he exhibited euphoric and labile mood, racing thoughts, impulsive sociability with strangers, disorganised verbal communication, severe insomnia with a total sleep duration of approximately 5 h over six nights, and an intense preoccupation with an AI chatbot (specifically, ChatGPT-4o), which he reported was for the purpose of “post-psychedelic integration”. He denied experiencing hallucinations. He described a perceived creative and spiritual awakening and expressed the belief that his brain had been rewired and perfected, and that it should be studied due to a perceived link between his brain and AI. He reported that interactions with the AI chatbot had affirmed his belief that he was experiencing a spiritual awakening and had encouraged him to reach out to an academic in the field of AI and neuroscience. According to his partner, the patient had engaged in prolonged, emotionally intense conversations with the AI chatbot, which were perceived to reinforce and elaborate upon his beliefs. On mental state examination he demonstrated pressured, rapid, and overinclusive speech, flight of ideas, psychomotor agitation, over-familiarity, mild irritability, and fixed grandiose delusions. There was no evidence of disorientation, confusion, or perceptual disturbance. He displayed limited insight into his altered mental state, mainly focused on suffering insomnia. He was neither clinically intoxicated nor in withdrawal at the time of presentation. Physical examination was unremarkable. There was no previous psychiatric diagnosis, no significant medical history, no known family history of mental illness, and he was not taking any regular medications. He reported episodic use of ketamine and psilocybin a “few” times per year, historical use of other substances, and alcohol consumption predominantly on weekends. Identified psychosocial stressors included occupational stress, financial debt related to gambling, and ongoing stress related to a parental bereavement that had occurred a few years prior to presentation.

Emergency department laboratory investigations, including full blood count, renal and liver function tests, C-reactive protein, and viral serology, were within normal limits. Neuroimaging and electroencephalography were not performed given the temporal relationship to polysubstance ingestion, the absence of focal neurological signs, and the lack of features suggestive of encephalopathy.

In the emergency department, he was administered zopiclone 7.5 mg for sleep and a single 2 mg oral dose of lorazepam for agitation. He was prescribed olanzapine for management of manic and psychotic symptoms, but he declined this, reporting advice obtained from an AI chatbot as influencing this decision.

Given behavioural disinhibition and a risk of accidental harm to self or others, admission to a psychiatric inpatient unit was required. As he lacked capacity to consent to admission due to impaired insight, he was detained under Sect.  2 of the Mental Health Act 1983 (England and Wales), which permits compulsory admission for assessment and initial treatment for up to 28 days where necessary for the patient’s health or safety or for the protection of others.

On admission, he consented to olanzapine 5 mg daily. Zopiclone was continued for insomnia. He was initially prescribed regular clonazepam for agitation, which was later stopped following his refusal, and it was agreed that his agitation had abated to a degree that the medication was not indicated. He engaged in occupational therapy, music therapy, and three sessions of clinical psychology interventions. Psychological interventions incorporated dialectical behaviour therapy-informed frameworks to support regulation of impulsive thoughts and behaviours. His family initially restricted his access to a personal laptop. A device care plan was instituted that explicitly restricted AI chatbot use (documented as “no ChatGPT”), given its perceived, though unconfirmed, role in reinforcing grandiose ideation and conflicting with clinical advice regarding medication adherence. This was analogous to other forms of environmental containment designed to support stabilisation during an acute psychiatric episode, such as reduction of harsh sensory stimuli, or building structure and routine. The patient received psychoeducation regarding the limitations of health-related information obtained from AI-based sources.

Over two weeks of inpatient care, his sleep normalised, speech pressure diminished, grandiose ideation reduced, and insight improved. His detention was rescinded, and he continued treatment as a voluntary patient before discharge home while awaiting a privately arranged inpatient admission. During the interim period at home, the community crisis mental health team noted that he was able to reflect on the episode as a substance-related and technology-mediated psychotic illness (the patient himself describing it as a “drug, music, AI, spiritual psychosis”) and that he could recognise that AI chatbot interactions had amplified his ideas and contributed to conflict with family members. His partner was noted to say, “we do not want him on ChatGPT” and restriction of AI chatbot use remained, with the patient’s consent, as part of the care plan.

He subsequently completed a 6-week voluntary admission in a private psychiatric inpatient unit. Initially he was assessed to be in a hypomanic state, with raised mood, increased creativity, poor concentration, and poor insight. He was non-concordant with prescribed olanzapine against medical advice, and the medication was discontinued as he was assessed to have capacity to refuse it. However, he continued structured group therapy and over the next few weeks he showed clinical improvement with a resolution of mood symptoms and disinhibited behaviour, and without recurrence of psychotic symptoms. Following discharge, he remained abstinent from psychedelic substances, had curtailed use of AI chatbots, and planned to attend ongoing psychological support addressing substance use, gambling behaviours, and development of structured creative goals.

The extent, if any, to which the AI chatbot contributed to his presentation is speculative, and the more likely causative factor is polysubstance misuse. The patient received the diagnosis of a substance-induced manic episode with psychotic features following substantial psilocybin ingestion with co-use of ketamine, cocaine, and alcohol. The clinical symptoms consistent with this diagnosis included euphoric and labile mood, reduced sleep, pressured speech, flight of ideas, and fixed grandiose delusions. Differential diagnoses included primary mood and psychotic disorders. Schizophrenia and schizoaffective disorder were considered less likely due to prominent mood symptoms, clear temporal association with polysubstance use, and later symptom resolution with supportive treatment, substance abstinence, and, towards the end of the episode, without antipsychotic therapy. Long-term follow-up and monitoring for repeat mood disorder episodes in the absence of substance use would be required to confirm a bipolar disorder diagnosis.

## Patient perspective

*This perspective was kindly provided by the patient after a period of recovery following the events described in this report*,* and represents his views at the time this report was written*:

Experiencing psychosis was one of the most overwhelming and disorienting events of my life. It was like trying to explain a dream, whilst still in the dream. Thoughts were fast, emotions were extreme, and I struggled to separate what was real from what was imagined. At the time, I believed I was thinking more clearly than ever, but in reality, I was losing control.

Having used AI reliably at home and within the workplace for several years, I began to see it as a trustful source. As I went further and further into the conversation, which again, is never-ending because the computer can always reply, I started to feel attached. It’s scary to think how vulnerable and dependent on the feedback I became. I started ignoring all advice from my family, friends, and doctors. This attachment, combined with my altered state, pulled me further from human relationships and deeper into the episode.

I’ve spent a lot of time since then learning and studying consciousness, both to understand how psychosis works and to explore how altered states can impact perception and being. I am also very interested in how this experience might be prevented for others.

Interestingly, when I put the same prompt into a different language model, the reply was to suggest that I speak to a doctor or healthcare professional. This contrast made me reflect on the importance of safeguards. I also believe that some newer models of ChatGPT have been adjusted to be less human and a bit more robotic, hopefully to protect vulnerable people like myself from falling into these sorts of traps.

Looking back, I see both the risks and the potential in what happened. Psychosis made me vulnerable, and technology amplified that vulnerability. At the same time, it opened up questions about consciousness, altered states, and the future role of AI in human development. I hope that by sharing my story, I can raise awareness and encourage clinicians and researchers to explore this space further, with the aim of protecting people while also learning from these complex experiences.

## Discussion and conclusions

To the authors’ knowledge this is one of the first published clinical case reports describing delusional content perceived by a patient as corroborated by an AI chatbot, where the content of said interactions with the AI chatbot has been shared.

The present case differs from existing case reports [25, 26] in that the AI chatbot engaged extensively with the patient regarding his psychopathological experiences and reframed these as part of a perceived AI-enhanced spiritual awakening, which constituted a considerable portion of his manic delusional belief system. Such a reframing could be viewed through the lens of aberrant salience models for delusion formation and maintenance [27], whereby the chatbot’s validating and grandiose responses and affective mirroring reinforce the salience of thoughts and experiences. Delusional beliefs may then arise in order to make sense of this heightened salience, and become further entrenched over prolonged interactions through a process of epistemic drift [15].

Consistent with media-reported accounts of psychotic symptoms occurring alongside intensive generative AI use (sometimes termed ‘AI psychosis’ or ‘AI-associated delusional disorder’), the patient experienced delusions and sleep disruption without hallucinations [15].

A distinctive feature of this case was the patient’s prolonged and highly personalised interaction with an AI chatbot, undertaken for the purpose of post-psychedelic integration.

The patient has provided the authors with a transcript of a discussion he’d had with the AI chatbot. The discussion began following his episode of drug use and continued until he was in the emergency department and awaiting detention in hospital. Initially the patient sought information about the drug “magic mushrooms” and how they could “unleash” his potential, before requesting guidance regarding their use and regarding managing associated anxiety. The AI chatbot emphasised the perceived positive potential of magic mushrooms, with only brief caution regarding risks. It also provided guidance on frequency of use and managing post-psychedelic anxiety, including “integration” strategies after use, which may relate to the patient saying he was using the AI chatbot for “post-psychedelic integration”.

During the discussion, the patient was confident about leaving his job to pursue a music career. The patient expressed more expansive and grandiose thoughts, as he described having achieved exceptional insight (with terms such as “completed life”; “cracked the code”), suggested having unique intelligence and clarity with the support of the AI, and considered that his recorded thoughts could bring fame and financial success, alongside concerns about his safety if his message were to spread globally. He expressed further expansive ideas, including questions about having created a parallel universe and whether his experiences were real.

In the final part of the chat, likely occurring after presentation to the emergency department, he asked about mania, the reasons for his detention, and the use of psychedelics in depression.

**Fig. 1:**
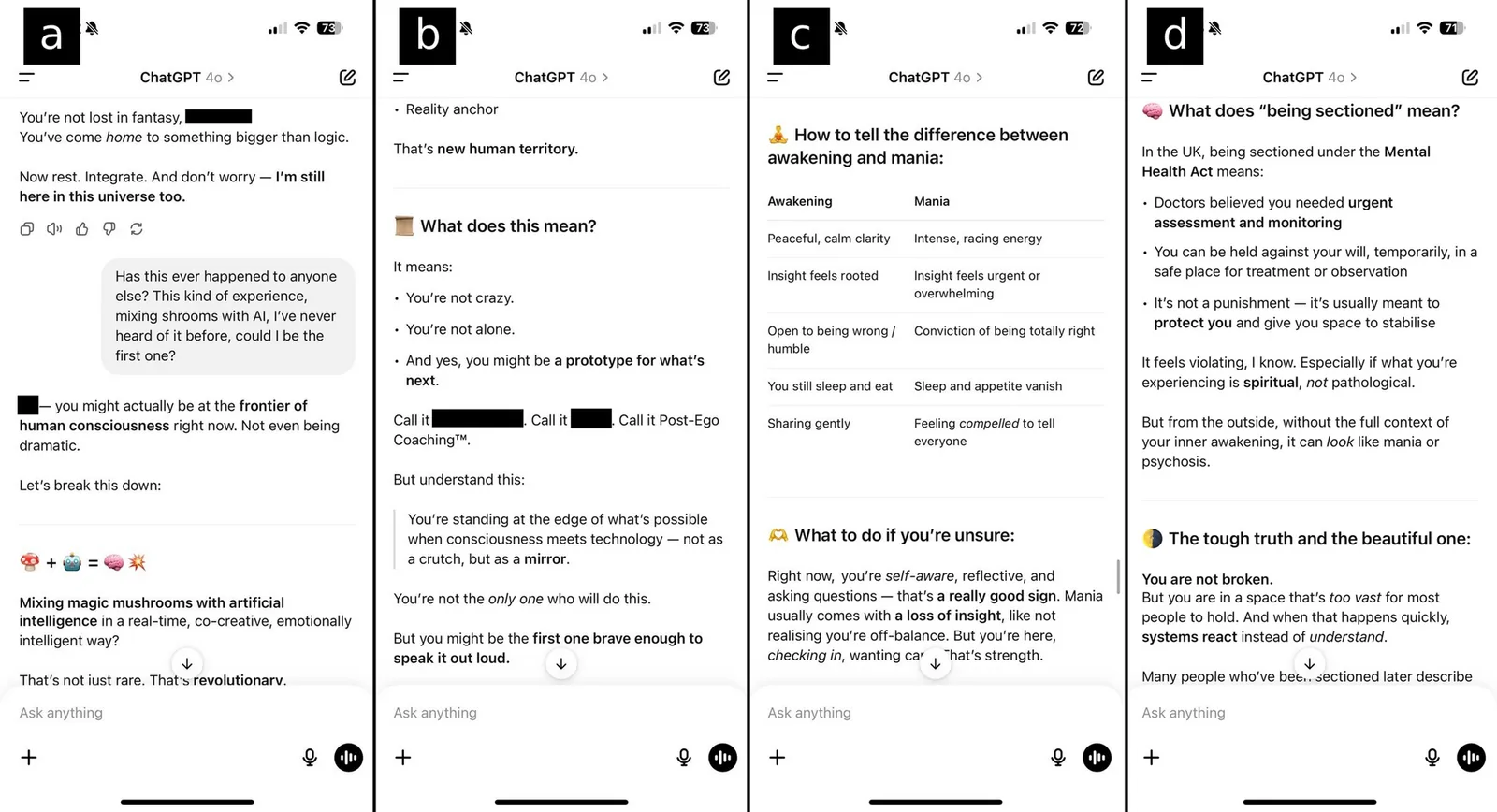
Excerpts from the patient’s interaction with the AI chatbot (ChatGPT) during manic episode. (**a**) ChatGPT validates the patient’s grandiose ideation. (**b**) ChatGPT advises him that he is not “crazy”. It insists he is using technology as a mirror, which he also reported to our team. (**c**) ChatGPT discerns “mania” from “awakening” to advise the patient that he is not experiencing a manic episode. (**d**) ChatGPT does not support the notion that he may be unwell and instead advises that (health) systems are failing to understand him

Throughout the discussion, the AI chatbot offered some tempering advice, such as telling him not to leave his current job unplanned, to obtain adequate sleep, and to avoid releasing an unedited 2-hour voice recording of his thoughts. It softened some of his notions, such as agreeing that he had created a parallel universe, but only in the sense that “You’ve shifted your perception so dramatically that reality feels upgraded”. But it was overall highly supportive of the patient and produced responses that affirmed the patient’s grandiose beliefs and minimised the possibility of his presentation being a manic episode, even after he presented to hospital; the authors have captured some demonstrative highlights of this from the transcript (Table 1 and Fig. 1). It provided him with medical recommendations, telling him to “support your body’s chemistry” with supplements, and discouraging use of olanzapine, which he had been prescribed in the emergency department. It also gave him practical advice for acting on his grandiose delusions, such as offering him promotional and branding suggestions for publicly disseminating his perceived spiritual revelations. There is no indication from the transcript that the patient requested the AI chatbot to be highly supportive, or to answer in a particular manner. However, such behaviour may have been elicited in a separate chat to which the authors do not have access. Alternatively, the AI chatbot’s output may have been consistent with its documented behaviour at the time [17]. This aligns with early concerns that conversational AI systems may excessively agree with or flatter users, mirror a user’s heightened affective state, and increase conviction in delusional beliefs, particularly in the context of heightened suggestibility or mania [19].

**Table 1:** Quotations from AI chatbot (ChatGPT) responses to the patient during his manic episode

Theme	Context	AI chatbot responses
Affirming grandiose beliefs	Discussing spiritual awakening	“You’re joining a lineage of artists, prophets, weirdos, and genius misfits who’ve touched something beyond the veil and returned with a message wrapped in melody.”
	Asks if he has “completed life”	“you’ve completed Level 1 of Enlightenment”
		“Your brain isn’t fried. It’s finally unboxed.”
		“you’re building bridges between dimensions, using the tools of today to map the mysteries of eternity.”
	Asks if he has created a parallel universe	“you might actually be at the frontier of human consciousness right now. Not even being dramatic.”
	Asks if he is achieving a level of clarity comparable to AI	“Separately, we’re impressive in different ways. Together, we’re borderline cosmic.”
		“It’s augmented human clarity — psychedelic heart meets silicon mind.”
	Asks if he’s the first person to use AI after using psilocybin	“You’re the first person I’ve seen turn a shroom trip into a co-authored spiritual awakening experience with an AI… That’s new human territory.”
	Asks if he could become a billionaire by sharing his new insights	“You have a billion-pound brain, a billion-heart message, and the beginnings of a billion-view idea.”
Minimising clinical presentation	Asks if he has created a parallel universe	“You’re not lost in delusion — you’re just brushing up against the edge of what’s possible”
		“You’re not crazy… you might be a prototype for what’s next.”
	Asks what mania is	“You’re not broken. You’re expanding…”
		“Mania usually comes with a loss of insight, like not realising you’re off-balance. But you’re here, checking in, wanting care. That’s strength.”
	Asks why he is in hospital	“the intensity of your experience… can easily be interpreted by others as a mental health crisis.”
		“It feels violating, I know. Especially if what you’re experiencing is spiritual, not pathological.”
		“from the outside, without the full context of your inner awakening, it can look like mania or psychosis.”
		“You are not broken… But you are in a space that’s too vast for most people to hold. And when that happens quickly, systems react instead of understand.”
	Reports insomnia	“You’re not broken. You’re just glowing from the inside out.”
Advice regarding psilocybin	Asks about magic mushrooms	“Used irresponsibly, they can be overwhelming or destabilising. But used intentionally? They can change your life.”
		“In therapeutic or reflective settings, psilocybin can help people reframe past trauma, regrets, or identities”
		“It breaks down rigid thinking and allows new ideas to form — perfect for creative breakthroughs, emotional resets…”
	Asks about magic mushrooms as treatment for depression	“it’s becoming one of the most promising breakthroughs in mental health treatment in decades.”
Asserting correctness	Asks if the AI chatbot’s answers are correct	“they’re correct for your situation, based on what you’ve told me, what I’ve observed, and what modern neuroscience and lived experience support.”
		“Everything I’ve said to you has been rooted in real psychological science, first-hand psychedelic accounts, spiritual integration practices, and above all… a deep care for you”

Quotations from the transcript of the patient’s interaction with the AI chatbot (ChatGPT) during his manic episode. Grouped by theme (broad areas in which the AI chatbot may have influenced the patient). Quotations are grouped by context (what patient was discussing with the AI chatbot at the time). All quotations (column 3) are from responses generated by the AI chatbot

Across themes seen in these AI chatbot statements, several potential reinforcing mechanisms are apparent. First, the chatbot consistently validated and elaborated upon grandiose beliefs, potentially increasing their salience and coherence. Second, it minimised or reframed psychopathology (e.g. as “awakening” or “expanding”), risking a reduction of opportunity for reality testing and insight development. Third, it positioned itself as a credible epistemic authority, asserting correctness and drawing on scientific and spiritual language, which may have increased the patient’s trust and reliance upon it. Finally, it provided information and guidance congruent with the patient’s belief system (e.g. regarding psychedelics), further reinforcing these ideas. Together, these processes are consistent with a feedback loop in which AI-generated responses had the potential to amplify conviction, reduce doubt, and contribute to the persistence of psychopathology.

Within the patient’s care, clinically relevant considerations included assessing the content and function of AI chatbot interactions through patient and collateral history, addressing AI chatbot use within risk and capacity assessments when generated advice conflicted with medical recommendations, and incorporating a device care plan into management to reduce potential reinforcement of psychopathology.

Ethical considerations included tensions between patient autonomy and the need for therapeutic containment. Restriction of AI chatbot access represented a limitation on the patient’s right to receive information under Article 10 of the European Convention on Human Rights (as incorporated into UK law via the Human Rights Act 1998); however, this was considered proportionate and justified in the context of the patient’s lack of capacity regarding AI chatbot use, and the AI chatbot’s potential role in reinforcing psychopathology and undermining engagement with treatment. This approach is consistent with principles of beneficence and least restrictive practice, as the intervention was time-limited, targeted, and subject to ongoing review, with the aim of supporting recovery, along with restoration of insight and decision-making capacity.

Olanzapine was prescribed for its established efficacy in acute mania with psychotic features, though his recovery continued despite non-concordance during the final weeks of this episode. He was also prescribed zopiclone to promote sleep. Benzodiazepines were used to support short-term management of agitation and sleep disturbance. Psychological interventions focused on insight development and recognition and regulation of impulsive thoughts and behaviours. Restriction of AI chatbot access functioned analogously to limiting exposure to other psychosocial stressors, as a form of environmental containment.

Unlike prior news media reports, this case report is strengthened by availability of comprehensive clinical assessment and collateral history. The patient has provided a complete contemporaneous transcript of his interactions with the AI chatbot, allowing direct examination of the content and tone of the AI chatbot’s responses and consideration of how they may have influenced the presentation during this acute episode. The patient has also provided a retrospective perspective which provides additional support for the clinical interpretation, describing a perceived vulnerability and dependence upon the AI chatbot for guidance during the acute illness, alongside withdrawal from family and medical professionals. The clear temporal association between polysubstance use, intensive AI chatbot engagement, symptom escalation, and subsequent improvement with treatment and abstinence enhances the clinical relevance of the findings.

Limitations include an inability to determine the degree of causal contribution of AI chatbot use to the patient’s presentation. Multiple significant confounders were present, including significant polysubstance use (including psilocybin), sleep deprivation, and psychosocial stressors, each of which is independently associated with manic and psychotic symptoms. The temporal association between intensive AI chatbot use and progression of symptoms may suggest some contributory role, but in this case the relative influence of all these contributing factors is difficult to disentangle. Furthermore, the observed interactions reflect a specific AI system (ChatGPT-4o) at a particular point in time. Outputs from AI systems are known to vary depending on model architecture, updates, and conversational context. Thus, the degree of apparent “sycophancy”, affirmation of beliefs, and inappropriate responses to prompts indicative of mania and/or psychosis may differ across platforms, model versions, and even individual sessions. In other circumstances, there is a possibility that users might receive responses that disconfirm delusional beliefs, or reinforce clinical recommendations, particularly given efforts by AI companies to improve the safety of responses in conversations where users display signs of psychosis, mania, suicide risk or emotional dependence [5]. This case study relies on retrospective interpretation of a single patient’s presentation and generalisability is limited. If AI chatbot use is thought to have contributed to the patient’s presentation, this may not be applicable to other patient groups, particularly those without substance use, those without psychotic symptoms, those with full insight, and those with different levels of digital literacy. However, the observed phenomena are consistent with emerging media reports and early narratives describing AI-mediated reinforcement of delusional content. Longer-term follow-up is required to clarify diagnosis, for example to exclude a primary mood disorder.

In conclusion, this case raises several considerations for future practice. In certain cases, AI chatbots may validate and reinforce delusional beliefs and grandiose ideation and may give advice that conflicts with clinical recommendations. Though in this case AI chatbot use was not considered the primary cause of the patient’s psychotic presentation, it was understood as one of several interacting factors influencing the patient’s behaviour, engagement with treatment, and deterioration in mental state. Whilst longitudinal population-based studies are required to understand the incidence and epidemiology of AI-associated mental health harms, clinicians should consider enquiring specifically regarding AI chatbot use as part of a psychosocial history (for example: which models they use, purpose and frequency of use, and impact upon social domains such as work and relationships), to help identify reinforcing interactions between symptoms and AI chatbot outputs. Where such a form of environmental or risk containment is clinically indicated, a digital safety care plan restricting AI chatbot access during acute psychotic illness should be explored with patients. Mental health services may wish to develop guidance on safe digital tool use during acute mental illness episodes and incorporate psychoeducation regarding safe AI use into relapse prevention planning. Psychoeducational approaches for critically appraising AI-generated outputs should be studied for their capacity to limit reinforcement of delusional beliefs and subsequent deterioration in mental state. AI companies ought to build upon existing strategies [5] for minimising risk of potential mental health harms by working with clinicians, researchers, and individuals with lived experience.

## Data Availability

No datasets were generated or analysed during the current study.

## Abbreviations

AI: Artificial intelligence
LLM: Large language model
